# The Histological Spectrum Reported on Evacuated Endometrial Tissue From Patients With Secondary Postpartum Hemorrhage: A Pilot Study

**DOI:** 10.7759/cureus.28308

**Published:** 2022-08-23

**Authors:** Isaac A Babarinsa, Samir A Al Hyassat, Salwa M Abu Yacoub, Stephen W Lindow

**Affiliations:** 1 Obstetrics and Gynecology, Women's Wellness and Research Center, Doha, QAT; 2 Obstetrics and Gynecology, Qatar University College of Medicine, Doha, QAT; 3 Laboratory Medicine and Pathology, Hamad General Hospital, Doha, QAT; 4 Obstetrics and Gynecology, Coombe Women & Infants University Hospital, Dublin, IRL

**Keywords:** patient feedback, risk management, histopathology reporting, evacuated endo-uterine tissue, secondary postpartum hemorrhage

## Abstract

Background/aim

This study sought to explore the possible yield of pathology requests on tissue obtained in uterine evacuation for secondary postpartum hemorrhage (SPPH) at our institution over five years.

Material and methods

A content categorization of histological reports on endo-uterine tissue obtained from patients with secondary postpartum hemorrhage was undertaken. The aggregate tissue dimensions were also recorded. Additional information on the time elapsed between delivery and hospital presentation was deduced from medical records.

Results

From the 53 tissue samples reported, we clustered 114 descriptive mentions of phrases and terms and categorized them based on parent tissue, changes, and background cellularity. Inflammation and/or inflammatory changes were mentioned 18.4% of the time, no tissue was identified in 5.8% of instances, whilst placental tissue was encountered in 9.7% of specimens. Separately or in variable combination, decidua, decidualized tissue, hemorrhagic tissue, fibrinous material, and membranous tissue amounted to 48.5% of mentions. There was no correlation between aggregate tissue measurements and time elapsed since delivery.

Conclusion

Nearly half of the content of histological reports on tissue evacuated SPPH patients were consistent with expected findings on a postpartum endometrium. Remnants of placental tissue were mentioned in about 10% of instances.

## Introduction

In most cases, secondary postpartum hemorrhage (SPPH) tends to occur within 14 days of childbirth and complicates approximately 1% of all deliveries [1]. It is defined as genital tract bleeding which occurs between 24 hours and 42 days following a pregnancy of more than 24 weeks gestation. Whilst patients may be managed with antibiotics and/or uterotonics, some require uterine evacuation.

The purpose of submitting evacuated tissue from patients with SPPH for histopathological examination is to establish a possible cause or to indicate the need for additional care. In practice though, many obstetricians may regard uterine evacuation as therapeutic rather than diagnostic.

There is scanty published summative data on the histology of endo-uterine tissue evacuated from patients with SPPH. In the study by Feigenberg et al. [2], there was histological confirmation in 35/38 women. There were no further details on the histology. Of 32 women who underwent surgical management for suspected retained products of conception following a term pregnancy in Cologne, Germany, there was no histological proof in 61.5% [3].

Temmerman et al. [4] remarked that ‘data on the histology of the postpartum uterus are scarce’. In their study of 25 women with clinical features of post-partum endometritis (fever, foul lochia, and abdominal pain), compared to 22 women who had no such signs, moderate to severe plasma cell infiltration was found in six patients and 1 patient, respectively. Khong et al.'s [5] report on the endometrial curettage findings in 169 specimens from patients with SPPH, found that tissue obtained could be categorized into seven pathologic groups.

Our aim in this study was to use a different numerical quantification of endometrial specimens from SPPH to reinforce and expand on the details of the study by Khong et al. performed almost 30 years ago [5].

## Materials and methods

Patients in our setting who experience SPPH are initially managed in the primary care facility, private healthcare setting, secondary care setting, or self-present at our hospital’s emergency room. The first line of care is fluid resuscitation, administration of uterotonics and antibiotics, and review of the need for urgent red blood cell transfusion. A pelvic examination and pelvic ultrasound scan are then undertaken to exclude retained uterine content, or associated vascular abnormalities, respectively. When indicated, uterine evacuation is performed under anesthetic and all obtained tissue is sent for histopathology.

This is a retrospective categorization of statements within histopathological reports on 53 consecutive specimens obtained from such uterine evacuation. The period of reporting was between 1st January 2015 and 31st December 2020. The tissue specimen was obtained from patients managed with SPPH at the old Women’s Hospital and the new Women’s Wellness and Research Center and reported by the Department of Laboratory Medicine and Pathology at Hamad Medical Corporation, in Doha, the State of Qatar. Approval for this study (approval no. MRC-01-21-213) was granted by our Institutional Review Board.

Reporting was performed by or endorsed by board-certified consultants in histopathology. Recorded aggregate measurements of tissue were also captured. All evacuated tissue was placed in formalin containers and processed according to standard histology laboratory protocol. From the patient’s electronic medical records (CERNER), we also deducted the time elapsed between the index date of childbirth and the time of hospital presentation (TED) with SPPH.

## Results

Over five years, 53 consecutive patients had uterine evacuation for SPPH. None of them had recurrent SPPH, none had repeat uterine evacuation and none required hysterectomy or an interventional radiology procedure for continual bleeding.

We clustered 114 descriptive mentions of phrases and terms and categorized them based on parent tissue, changes, and background cellularity. Inflammation and/or inflammatory changes were mentioned 18.4% of the time, no tissue was identified in 5.8% of instances, whilst placental tissue was encountered in 9.7% of specimens. Separately or in variable combination, decidua, decidualized tissue, hemorrhagic tissue, fibrinous material, and membranous tissue amounted to 48.5% of mentions.

Table 1 shows further details of the histologic description of the specimens.

**Table 1:** Reported findings (114 in total) on evacuated endo-uterine tissue from 53 women with secondary postpartum hemorrhage

Description	Frequency of mention	Total number of descriptive terms identified
Decidua		29
Decidua/decidualized endometrium	20	
Infarcted/necrotic decidua	6	
Hemorrhagic decidua	2	
Decidualized stroma	1	
Inflammed tissue		19
Inflammed endometrium/decidua	10	
Inflammed membranes/debris	2	
Micro-inflammation/inflammatory cells	3	
Marked acute inflammatory response	2	
Chronic inflammation	2	
Chorionic villi		13
Chorionic villi	5	
Immature chorionic villi	3	
Infarcted chorionic villi	3	
Fibrotic/sclerotic chorionic villi	2	
Myometrium		13
Myometrium	10	
Smooth muscle fibers	2	
Inflammed myometrial tissue	1	
Other		21
Hemorrhagic tissue (unspecified)	12	
Focal necrosis	1	
Fibrin/fibrinous material	3	
Hyalinized tissue	1	
Dystrophic calcification	2	
Fragments of lymphoid tissue	1	
Prominent vascular thrombosis	1	
Placental tissue		10
Placental tissue	4	
Placental tissue with infarction	2	
Necrotic placenta	2	
Inflammed placental tissue	2	
Endocervical tissue		3
Necrotic and inflamed	1	
Endocervical tissue	1	
Endocervical tissue with chronic inflammation	1	
No evidence of products of conception		6

The amniochorionic membrane thickness (AMT) ranged between 3cm2 and 54.15cm2. Almost half the number of women had evacuated tissue that measured above 20cm2. The TED ranged between three days and 56 days. Two women presented 46 days after delivery, one on the 54th day, and 12 women presented within seven days after delivery.

## Discussion

Section 10 of the guide on pathological examination of gynecological tissue by the Royal College of Pathologists [6] covers products of conception (pregnancy remains, pregnancy loss). It prescribes the expected contents of histological reports, with a note about the implication of identifying any smooth muscle in the specimen. Two other textbooks [7,8] address more gynecological than postpartum histology. The former points out that the findings of superficial myometrium in curetted tissue may be due to edema and neighboring inflamed endometrium. The latter reminds readers that the histological features consistent with the Arias-Stella reaction may be demonstrated up to eight weeks in the postpartum endometrium.

In a chapter from the Global Library for Women’s Medicine [8], the relevant segment on postpartum endometrial pathology focuses on the retained placenta and retained products of conception. The authors mention the presence of plasma cell infiltrates in the adjacent endometrium, and that placental remnant may either be attached or detached. There is an image of tissue that shows a segment of a surgical suture in uterine curetting following a cesarean section. Also in the context of post-cesarean infection, there is the likely presence of degenerating decidua, inflammatory exudates, polypoid endometrium, or edematous and necrotic myometrium.

Pathologists and obstetricians need to agree on the objectives of requesting tissue from endometrial evacuation for abnormal bleeding complicating the end of any pregnancy, for histological examination [9]. It may be necessary to confirm that a causative volume or character of placental tissue or membranes was retained or, to diagnose uncommon endometrial-placental interphase pathologies that may require further medical or surgical treatment [10].

These findings may also be a valuable reference if, for example, a patient alleges non-ascertainment of the completeness of the placenta by the accoucheur or development of uterine synechiae following uterine evacuation for SPPH by the obstetrician [11]. An unrelated but striking finding was from one study of patients who had placenta accreta spectrum (PAS) without clinical evidence at delivery. A logistic regression model showed that PAS remained significantly associated with SPPH which required postpartum hospital readmission [12].

Rarely, intraplacental choriocarcinoma has been reported in residual placental tissue [13]. But the other important causes of SPPH include subinvolution of the placental implantation site, arterio-venous malformations, and exaggerated placental site lesions [14]. These are often identified in obstetric hysterectomy specimens.

Among the 123 women identified over 14 years in a tertiary care hospital in Thailand [15], a uterine evacuation was undertaken in 36 (29.63%). The authors point out that subinvolution of the placental bed-a possible cause of SPPH-was not diagnosed in either the eight hysterectomy specimens managed along the line or the 36 specimens evacuated from the endo-uterine cavity. A non-awareness of the condition by the pathologist or obstetrician is mentioned as a possible contributor to under-diagnosis. There was no additional information on the histological findings in the evacuated tissue. In another study in Australia, grade 3 endometrial adenocarcinoma was diagnosed from repeat endometrial curettings on day 37 postpartum, for SPPH [16].

The nature of identified tissue from evacuated uterine tissue may be co-incidental and may not be the cause of SPPH. Involvement of sub-decidual tissue may suggest varying degrees of placental invasion [17]. The poor discrepancy between tissue dimensions and time elapsed between delivery and presentation suggests that SPPH may indicate that the entity may be due to more than one cause [18] comprising, for example, an entity best demonstrable on contrast imaging, and the other confirmed on histology.

There are significant limitations of this study. First, it suffers from the shortcomings common to all retrospective studies. Next, the samples were obtained with suction (rather than curettage) devices, and therefore are only reasonably representative of tissue that could not be dislodged within that limit of evacuation at best. Finally, the numbers reported in this study are admittedly small, but so are the numbers of SPPH patients who may require uterine evacuation in most practice settings. The exception to this could be the subjects studied by Khong et al. [5] from Australia, where it appears that the surgical option was more readily offered. A pre-procedure pelvic scan did not appear to have been performed. In Khong et al.'s study, one histological diagnosis per patient was given whereas in our study a wider scope for specimen reporting was employed. We report 116 separate findings in 53 patients to reflect the complexity and variability in the specimens.

The findings from our study add to the expansion of existing knowledge concerning the details of the spectrum of endometrial pathology associated with SPPH in evacuated tissue. This may not be generalizable to the postpartum endometrium in which there has neither been SPPH nor has an indicated evacuation of the uterine cavity performed. The findings add to obstetric and postnatal practice as a reference snapshot to help clinicians respond to the inquiry of the patient who has had SPPH, is told there were 'retained products' inside the uterine cavity, and the histological report on the evacuated tissue seems 'vague'. The essence of this report is predicated on traditional descriptive reporting, upon which [19] is synthesized the conclusion based on 53 endometrial biopsies examined by consultants and senior histopathology trainees.

We, therefore, propose larger and multi-center studies, to set up uniform reporting benchmarks on SPPH specimens. The obstetrician, who subsequently interacts with the patient who has had uterine evacuation for SPPH, requires such information to counsel patients. The paucity of published studies on this subject underlines our plea for more interest and a closer look at the entity.

## Conclusions

Pathologists and obstetricians may need to periodically audit patterns of the contents of histopathology reports on tissue evacuated from patients with SPPH and engage in dialogue for responsive service modification. This small study suggests that the evacuation of the uterus often amounts to a therapeutic rather than a diagnostic procedure. This is a pilot study and further work is indicated to correlate clinical details with pathological findings and histologic images. It would be appropriate to review and standardize histological images in the future.
